# Immobilization of TiO_2_ Nanoparticles on *Chlorella pyrenoidosa* Cells for Enhanced Visible-Light-Driven Photocatalysis

**DOI:** 10.3390/ma10050541

**Published:** 2017-05-17

**Authors:** Aijun Cai, Aiying Guo, Zichuan Ma

**Affiliations:** 1 College of Life Science and Technology, Hebei Normal University of Science & Technology, Qinhuangdao 066600, China; 2755@hevttc.edu.cn (A.C.); 2695@hevttc.edu.cn (A.G.); 2 College of Chemistry and Material Sciences, Hebei Normal University, Shijiazhuang 050016, China

**Keywords:** TiO_2_, visible light, photocatalysis

## Abstract

TiO_2_ nanoparticles are immobilized on chlorella cells using the hydrothermal method. The morphology, structure, and the visible-light-driven photocatalytic activity of the prepared chlorella/TiO_2_ composite are investigated by various methods. The chlorella/TiO_2_ composite is found to exhibit larger average sizes and higher visible-light intensities. The sensitization of the photosynthesis pigment originating from chlorella cells provides the anatase TiO_2_ with higher photocatalytic activities under the visible-light irradiation. The latter is linked to the highly efficient charge separation of the electron/hole pairs. The results also suggest that the photocatalytic activity of the composite remains substantial after four cycles, suggesting a good stability.

## 1. Introduction

Recently, visible-light-driven photocatalysts have attracted considerable attention for their possible utilization in energy conservation and environmental remediation. Therefore, the design and fabrication of efficient, cost-effective photocatalysts are increasingly investigated. TiO_2_ is considered as a classic photocatalyst, extensively studied for potential use in hydrogen energy [1,2], solar cells [3,4], and environmental cleaning [5,6], owing to its excellent stability, low toxicity, abundant quantities, and low cost. However, the highly efficient use of TiO_2_ has been limited by the quick recombination of its photogenerated electron/hole pairs, hence its inability to respond to the visible light. To overcome these problems, tremendous efforts have been devoted to enhancing its photocatalytic capacity. So far, traditional methods, including semiconductor coupling [7,8], metal-ion doping, anion doping [9,10], noble metal loading [11], and dye sensitization [12] have been explored.

On the other hand, the method based on bio-templating has recently attracted considerable attention in the synthesis of photocatalysts. Compared with engineered templates, natural biological materials are abundant, renewable, hierarchical, and environmentally benign [13,14,15,16]. With respect to this, numerous studies conducted by us led to the development of a series of bio-templating methods to prepare N-I doped ZnO [17], Cu hollow spheres [18], and ZnO/graphene quantum dots (GQDs) composites [19], among others.

Chlorella is classical single-cell green algae with sizes ranging from 3 to 8 μm, and is known for its photosynthetic ability and nutrition. It has abundant photosynthesis pigments, such as β-carotene and chlorophyll, thus resulting in high photosynthetic efficiency. Therefore, Chlorella is considered as a potential natural dye sensitizer source to produce dye-sensitized TiO_2_ with the ability to respond to the visible light.

In this paper, *Chlorella pyrenoidosa* cells were combined with TiO_2_ to prepare visible-light-driven TiO_2_ photocatalyst using an in situ dye sensitization method. The synthesized chlorella/TiO_2_ composite is then characterized by various techniques and its visible-light photocatalytic activities using rhodamine (RhB) as a model pollutant are examined.

## 2. Experimental

### 2.1. Materials

Analytical grade chemicals were used as received without further purification. *Chlorella pyrenoidosa* cells were purchased from Yueqing Biological Technology Co., Ltd. (Yueqing, China) Titanium tetrabutoxide (TBOT), acetonitrile, and ammonia were obtained from Tianjin Yong Da Chemical Reagent Development Center (Tianjin, China). Fluorine-doped tin oxide (FTO) conducting glass substrates were purchased from Asahi Glass Company (Tokyo, Japan).

### 2.2. Synthesis of Chlorella/TiO_2_ Composite

Typically, *Chlorella pyrenoidosa* cells (0.05 g) were dispersed in an 80 mL mixture of ethanol/acetonitrile (3:1 *v*/*v*). Under constant stirring, the dispersion was subsequently mixed with 0.6 mL ammonia. Next, a solution containing 1.2 mL TBOT in 20 mL of ethanol/acetonitrile (3:1 *v*/*v*) was added to the suspension. The mixture was then stirred for 2 h at room temperature, and the resulting precipitates were centrifuged and washed several times with deionized water.

Afterward, the as-obtained precipitates were re-dispersed in 20 mL deionized water and put in a Teflon-lined stainless steel autoclave with a volume of 30 mL at 180 °C for 10 h. After centrifugation and washing with deionized water, the chlorella/TiO_2_ composite was obtained.

Pure TiO_2_ as a control sample was also prepared using the same method but without *Chlorella pyrenoidosa* cells.

### 2.3. Characterization

The X-ray diffraction (XRD) patterns were recorded on a Bruker D8-ADVANCE (Bruker Co., Billerica, MA USA) X-ray powder diffractometer with Cu-Kα radiation. Transmission electron microscopy (TEM) images were obtained with a FEI Tecnai G2 F20 S-TWIN microscope (FEI, Hillsboro, AL, USA). Field-emission scanning electron microscopy (FESEM) images were recorded on a Hitachi S4800 elect ron microscope (Hitachi, Ltd., Tokyo, Japan). X-ray photoelectron spectroscopy (XPS) analysis was performed with an ESCALAB 250 instrument (Thermo VG Scientific, Waltham, MA, USA). The Brunauer–Emmett–Teller (BET) specific surface area and Barrett–Joyner–Halenda (BJH) analysis were carried out using a Micromeritics 3Flex surface characterization analyzer (Micromeritics Instrument Co., Norcross, GA, USA). A Hitachi U-4100 spectrophotometer (Hitachi, Ltd., Tokyo, Japan) was employed to acquire the UV-vis diffuse reflectance spectra (DRS), and BaSO_4_ was used as a reference sample.

The behavior of the photogenerated charge carriers in the samples was characterized by surface photocurrent (SPC) and electrochemical impedance spectroscopy (EIS), using our modified method [20]. The electrochemical measurements were acquired on a CHI-660B electrochemical system with a conventional three-electrode cell configuration. A Pt wire was used as a counter electrode and a saturated calomel electrode (SCE) as a reference electrode in an electrolyte solution of 0.5 M Na_2_SO_4_. The working electrode was prepared on a 20 mm × 33 mm FTO glass as follows: the FTO glass was firstly cleaned by sonication in acetone for 0.5 h and dried at 80 °C. The boundary of FTO glass was then covered with an insulating tape, with the exposed effective area of 1 cm^2^. Fifty milligrams of as-prepared products were suspended in 5 mL ethanol, which was then dip-coated on the pretreated FTO glass and annealed at 300 °C for 2 h. The variation in the photoinduced current density as a function of time (*i-t* curve) was measured at a bias potential of 0.5 V (vs. SCE) over five cycles during which the light was switched on and off. A 300 W Xe lamp with a 420 nm cutoff filter was utilized for the visible-light irradiation. The EIS test was carried out at the open-circuit potential with the frequency range from 0.1 to 10^6^ Hz.

### 2.4. Photocatalytic Activity Measurements

The photocatalytic activity of RhB degradation was measured using the following methodology: First, the catalysts (50 mg) were dispersed in 50 mL RhB aqueous solution (5 mg/L). Prior to visible-light exposure, the suspension was magnetically stirred in the dark for 30 min to reach the adsorption–desorption equilibrium. Under constant stirring, the mixture was then placed under a 500 W Xe lamp with a 420 nm cutoff filter. Next, using regular irradiation steps, aliquots (4 mL) were withdrawn from the suspension at intervals of 30 min. The RhB concentration change was monitored using UV-Vis absorption spectroscopy at wavelength of 553 nm. After dye degradation, the photocatalyst was centrifuged and washed several times with deionized water and anhydrous ethanol, respectively. The centrifugate was re-suspended in 50 mL RhB aqueous solution (5 mg/L), and used for the next cycle of dye degradation under visible-light irradiation. The RhB degradation efficiency (D%) is calculated using Equation (1):

D% = (1 − *C*/*C*_0_) × 100% (1)

where *C* and *C*_0_ represent the RhB concentration at time *t* and the equilibrium concentration of RhB, respectively [21].

The kinetics related to the degradation of RhB was also investigated, and the experimental data were fitted with the pseudo first-order-kinetic equation expressed by Equation (2):

ln(*C*/*C*_0_) = −*kt* (2)

where *k* is the apparent reaction rate constant (min^−1^).

## 3. Results and Discussion

**Figure 1:**
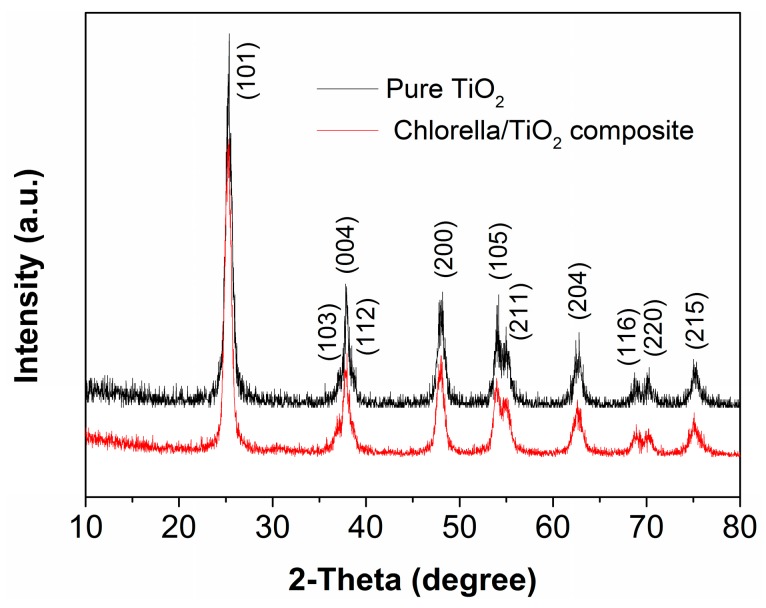
XRD patterns of pure TiO_2_ and chlorella/TiO_2_ composite.

The XRD patterns of pure TiO_2_ and chlorella/TiO_2_ composite are shown in Figure 1. The chlorella/TiO_2_ composite is ascribed to the anatase TiO_2_ phase (JCPDS card No. 21-1272), and exhibits a similar XRD pattern to pure TiO_2_. Both chlorella/TiO_2_ composite and pure TiO_2_ show similar full width at half maximum, suggesting similar grain sizes based on Scherrer’s equation [22,23].

**Figure 2:**
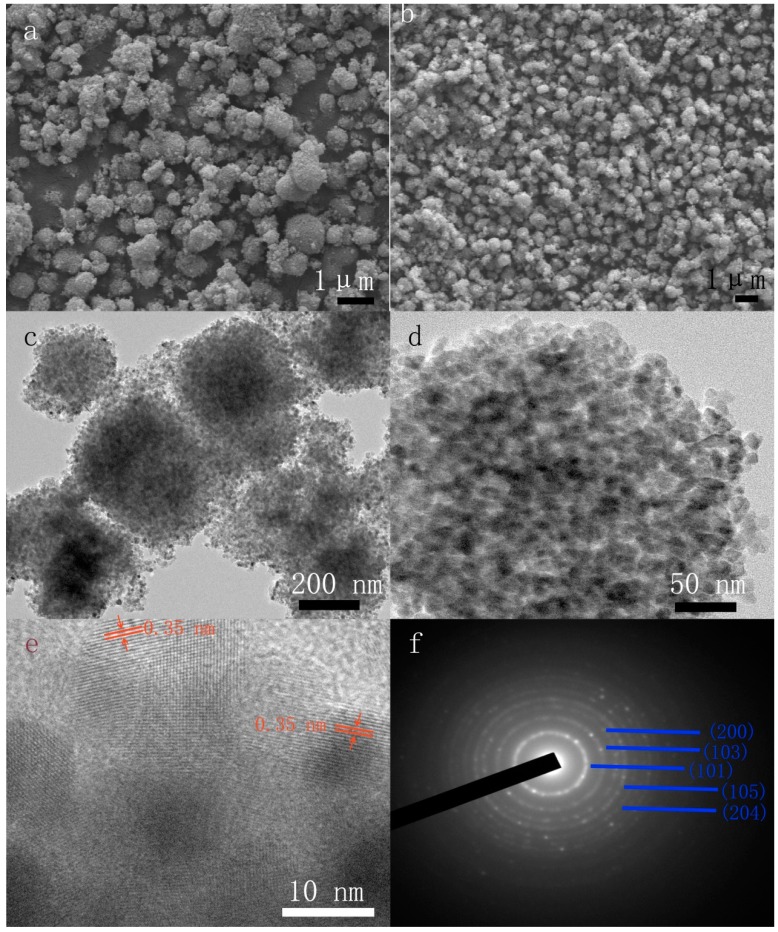
Field-emission scanning electron microscopy (FESEM) images of (**a**) the chlorella/TiO_2_ composite and (**b**) the pure TiO_2_; (**c**) TEM image of the chlorella/TiO_2_ composite; (**d**) Enlarged TEM image of a representative chlorella/TiO_2_ composite; (**e**,**f**) HRTEM image and SAED pattern of the chlorella/TiO_2_ composite, respectively.

The morphology of the chlorella cells is shown in Supporting Information (Figure S1). The chlorella cells are of sphere-like shape, and do not change after hydrothermal treatment. The morphology of the products was examined by FESEM and the results are depicted in Figure 2. As seen in Figure 2a, the overall image of the chlorella/TiO_2_ composite shows that the product exhibits a quasi-spherical shape. Although the pure TiO_2_ shows a similar spherical morphology to the chlorella/TiO_2_ composite (Figure 2b), the average size of the pure TiO_2_ is smaller than that of the chlorella/TiO_2_ composite. As exhibited in Figure S2, the average sizes of the pure TiO_2_ and chlorella/TiO_2_ composite are 620 and 790 nm, respectively. This result shows that the *Chlorella pyrenoidosa* cells play an important role in mediating the crystal growth of the products. The TEM images shown in Figure 2c,d reveal that the chlorella/TiO_2_ composite is composed of nanocrystals of about 12 nm in size. The HRTEM image estimates the *d*-spacing between two consecutive planes to be about 0.35 nm, agreeing well with the *d* (101) values of the anatase phase. The polycrystalline anatase phase is further confirmed by the selected area electron diffraction (SAED) pattern (Figure 2f), which is consistent with the XRD powder pattern.

**Figure 3:**
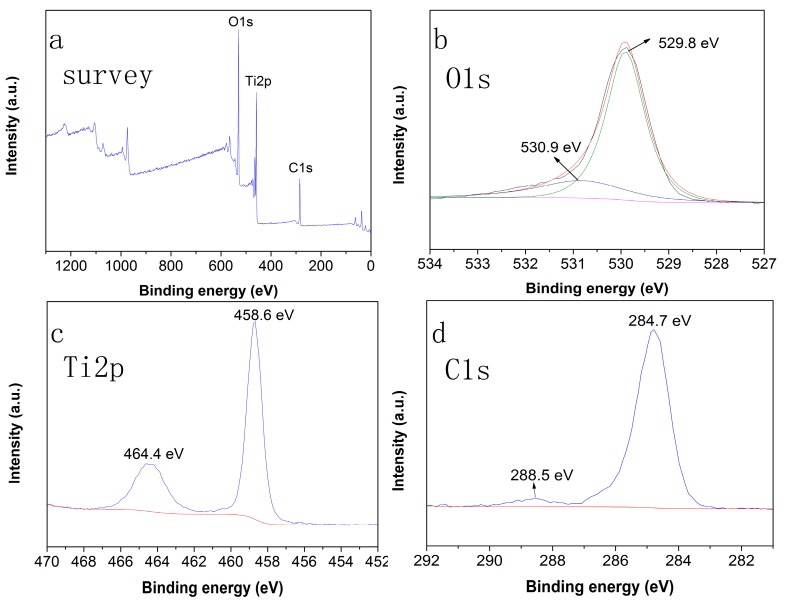
(**a**) XPS spectra of the chlorella/TiO_2_ composite; High-resolution XPS spectra of (**b**) C 1s; (**c**) Ti 2p; and (**d**) C 1s.

Figure 3 shows the XPS results for the chlorella/TiO_2_ composite. As shown in Figure 3a, the XPS survey spectrum illustrates the presence of signals of O 1s, Ti 2p, and C 1s. The XPS high-resolution core level spectra are shown in Figure 3b–d. The high-resolution scanning spectrum of O 1s displayed in Figure 3b shows two peaks: 530.9 eV and 529.8 eV. The peak at 530.9 eV can be attributed to the Ti–O binding [24], and the low binding energy component located at 529.8 eV could be due to the oxygen in the oxide lattice [25]. Figure 3c shows a Ti 2p XPS spectrum. Two peaks are observed at 464.4 eV and 458.6 eV, which are assigned respectively to the Ti 2p_1/2_ and Ti 2p_3/2_ spin-orbital splitting photoelectrons in the Ti^4+^ state in anatase titanium [26]. As shown in Figure S3, the binding energy of Ti in the chlorella/TiO_2_ composite does not exhibit evident changes, compared with that of the pure TiO_2_. As shown in Figure 3d, C 1s spectrum exhibits two peaks at 288.5 eV and 284.7 eV. The peak at 288.5 eV corresponds to the C=O from carbonyls and carboxylates, and the peak at 284.7 eV is attributed to the carbon from chlorella cells and/or contamination. Compared with the pure TiO_2_, the chlorella/TiO_2_ composite has higher C 1s content due to the richness of the carbon from chloral cells, which is shown in Table S1. However, the peaks corresponding to the Ti–C bond (282 eV), Ti 2p_1/2_ (466.0 eV) and Ti 2p_3/2_ (460.3 eV) were absent, indicating no carbon doping in the lattice of TiO_2_ [27].

**Figure 4:**
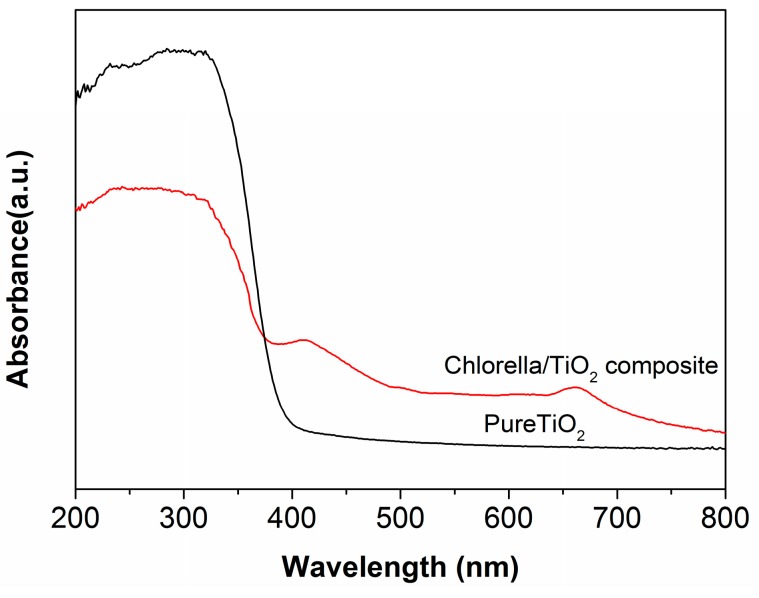
UV-vis diffuse reflectance spectra (DRS) of the chlorella/TiO_2_ composite and the pure TiO_2_.

Figure 4 shows the DRS results of the two samples. The pure TiO_2_ exhibits strong absorption in the ultraviolet region, due to the transition of the excited state electrons from the valence band (VB) to the conduction band (CB) under the UV irradiation. In comparison with the pure TiO_2_, the chlorella/TiO_2_ composite shows elevated visible-light absorbance intensities, which contributes to the improvement of the photocatalytic activity under visible-light irradiation.

**Figure 5:**
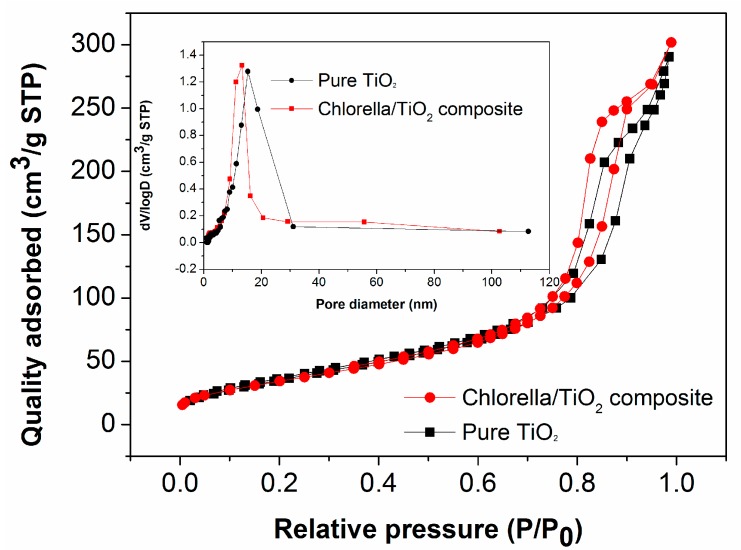
Nitrogen adsorption isotherms for the chlorella/TiO_2_ composite and the pure TiO_2_, respectively. The inset shows typical plots of the size distribution.

The N_2_ adsorption and desorption isotherms are shown in Figure 5. According to IUPAC classification [14], both the chlorella/TiO_2_ composite and the pure TiO_2_ should have similar isotherm curves (type IV) and H1 hysteresis loops. The surface areas of the chlorella/TiO_2_ composite and the pure TiO_2_ are estimated to be 138.5 m^2^/g and 130.1 m^2^/g, respectively. Due to the similar surface areas, it is conferred that the surface area should not be the primary factor devoted to the visible-light-driven photocatalytic performance enhancement. The mesoporous structures of both samples are further confirmed by the pore size distribution analysis, shown in the inset of Figure 5. The mesoporous peaks for the chlorella/TiO_2_ composite and the pure TiO_2_ are estimated to be 13.7 nm and 15.6 nm, respectively. Although the two samples exhibit narrowly distributed mesopores, the chlorella/TiO_2_ composite shows a macroporous peak at ca. 56.1 nm, which further contributes to the entrancement of the dye molecules during the photocatalysis.

**Figure 6:**
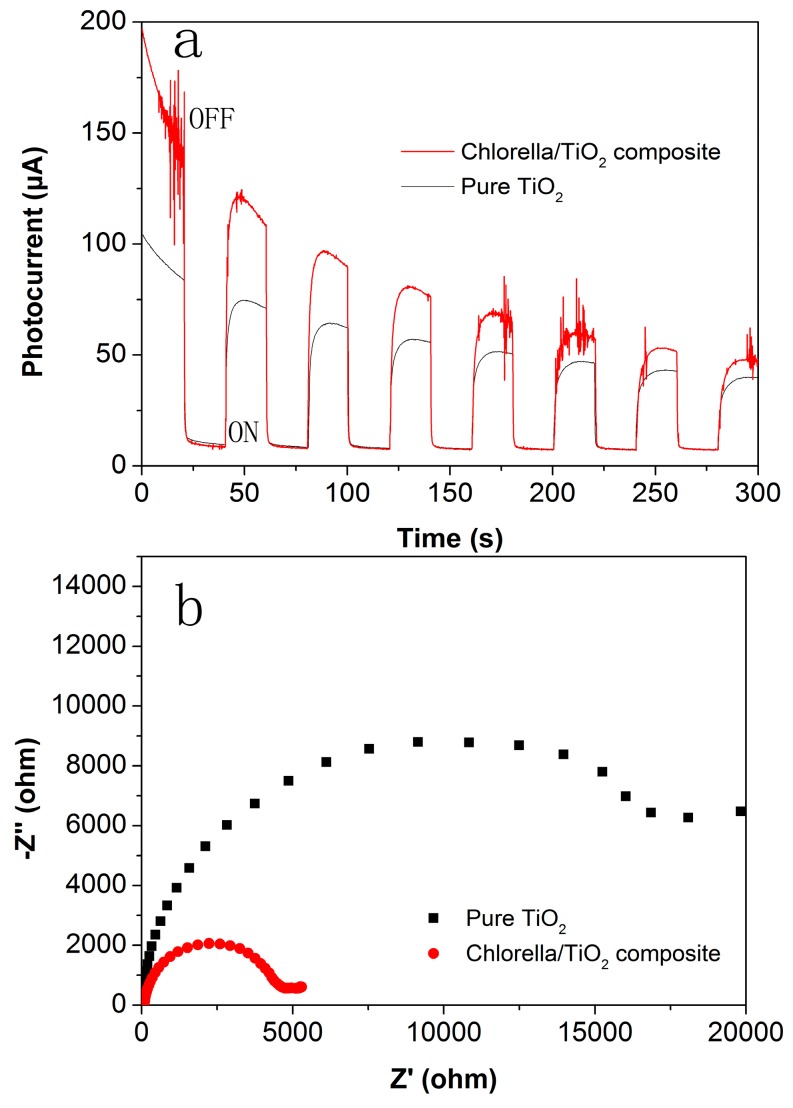
Surface photocurrent (SPC) (**a**) and electrochemical impedance spectroscopy (EIS) (**b**) results of the samples.

To study the separation and transfer behavior of the photogenerated charges in the visible region, SPC and EIS were performed and the results are shown in Figure 6. Figure 6a shows the result of the SPC of the samples. It can be seen that a steady and prompt photocurrent generation is observed in the two samples under visible-light irradiation. The chlorella/TiO_2_ composite exhibits a higher photocurrent density, compared with the pure TiO_2_, suggesting enhanced photoinduced separation of electron/hole pairs [28]. The EIS result of the samples is shown in Figure 6b. The semicircle in EIS Nyquist plot of chlorella/TiO_2_ composite becomes shorter than that of the pure TiO_2_, suggesting a decrease in the solid-state interface layer resistance and the charge transfer resistance on the surface [29]. The result indicates the effective separation of photogenerated electron/hole pairs and fast interfacial charge transfer in the chlorella/TiO_2_ composite.

**Figure 7:**
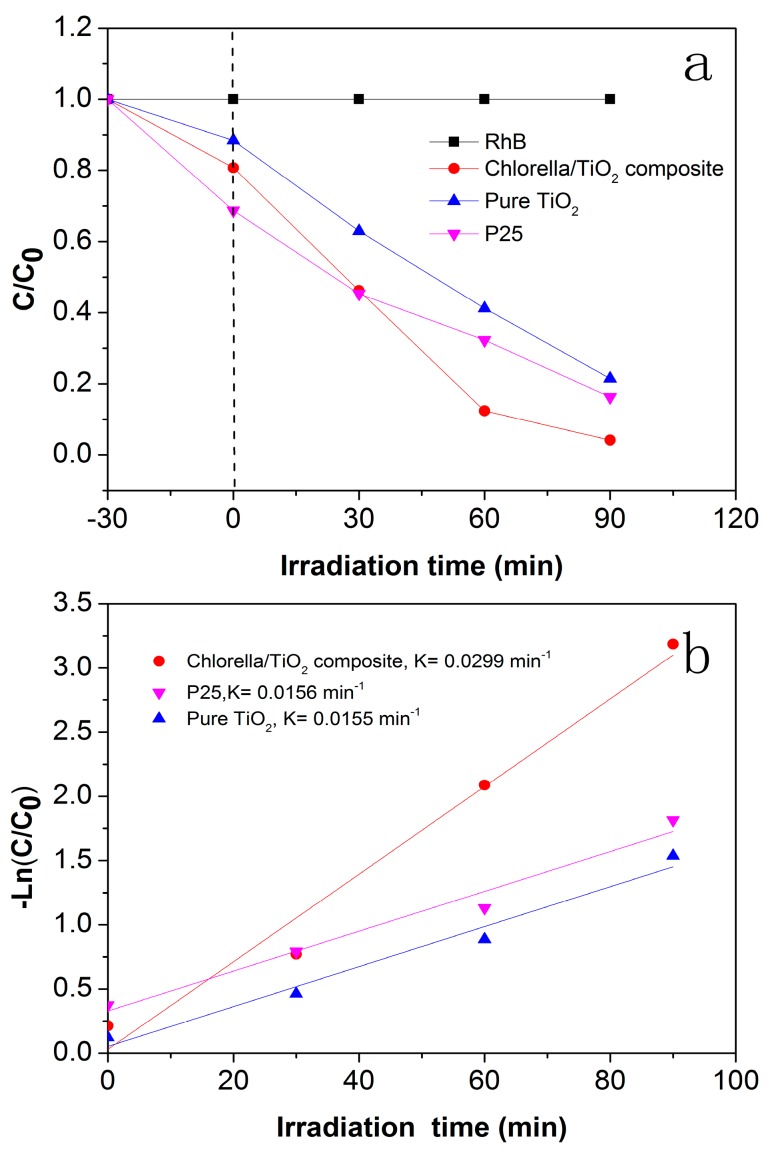
(**a**) Photocatalytic degradation of rhodamine (RhB) solution with the samples under visible-light irradiationhotocataly; (**b**) Photodegradation kinetics lines.

The photocatalytic activities were examined through the degradation of RhB solution under visible-light illumination. For comparison, the photocatalytic activity of commercial TiO_2_ (P25) was also tested under the same reaction conditions. As shown in Figure 7a, RhB dye cannot be degraded in the absence of the photocatalysts under visible-light irradiation, confirming the photostability of the dye. After 90 min irradiation with visible light, around 78.5% and 83.7% of RhB are degraded by the pure TiO_2_ and P25, respectively. While after the same time, 95.9% of the RhB is successfully degraded in the presence of the chlorella/TiO_2_ composite. The result demonstrates that the chlorella/TiO_2_ composite has the highest photocatalytic activity, due to the coupling of TiO_2_ with the biotemplates. Figure 7b shows the kinetics lines. From the slopes of the curves, the *k* for the chlorella/TiO_2_ composite, the pure TiO_2_ and P25 is determined as −0.0299, −0.0155 and −0.0156 min^−1^, respectively. The valid photocatalyst is TiO_2_ that is only part of the chlorella/TiO_2_ composite. Although the actual content of TiO_2_ in the chlorella/TiO_2_ composite is lower as compared with the pure TiO_2_, the chlorella/TiO_2_ composite is able to degrade the RhB much faster than another sample.

In addition, the hydrothermal treatment is found to play an important role in the preparation of the samples. It is well known that TiO_2_ exhibits an amorphous phase, using the sol-gel method of TBOT [30,31]. The chlorella/TiO_2_ composite and pure TiO_2_ can be transferred from amorphous to anatase phase (JCPDS card No. 21-1272) through the hydrothermal treatment, which is confirmed by the XRD patterns (Figure S4). As shown in Figure S5, the amorphous products reveal lower visible-light-driven photocatalytic activities for degradation of RhB.

**Figure 8:**
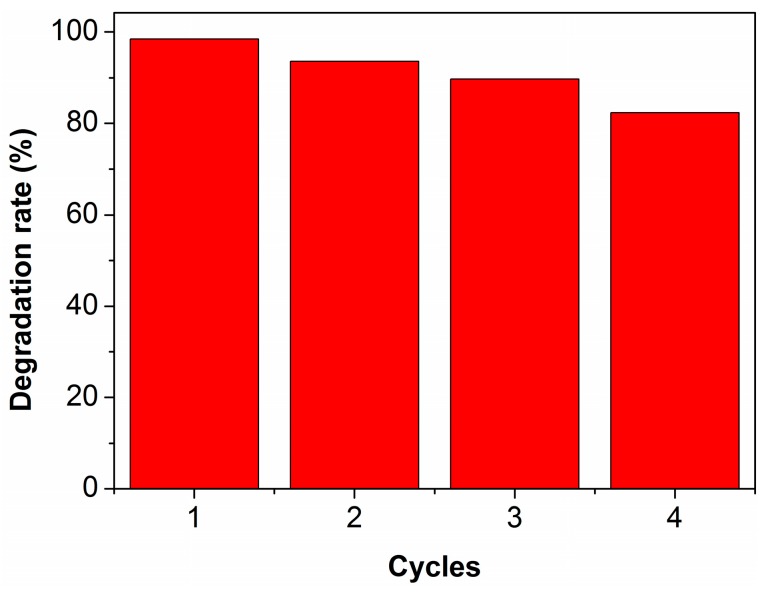
The photocatalytic stability of the chlorella/TiO_2_ composite towards the degradation of RhB.

A recycling experiment was also performed to verify the photocatalytic performance of the chlorella/TiO_2_ composite. As shown in Figure 8, the catalyst still exhibits high photocatalytic activity towards RhB with a degradation rate of 82.3% after the four cycles, indicating a good stability.

Zhang et al. synthesized spirulina/TiO_2_ composite to degrade methyl orange (MO) under visible-light irradiation [12]. They obtained a similar *k* value to ours for the composite, however, they have not compared P25 with the as-prepared composite, and the stability of the composite has not been determined. Our results confirm that the algae, such as spirulina and chlorella, can be used as an efficient sensitizing source to improve the visible-light photocatalytic activity of TO_2_. Moreover, there are abundant algae in the sea and freshwater, which presents a new method in mass-fabricating photocatalysts.

**Figure 9:**
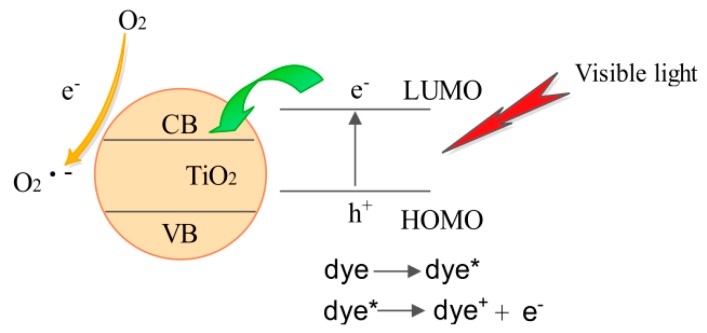
The mechanism of visible-light activation of TiO_2_ by dye sensitization.

Previous studies have shown that dye sensitization is a useful method to induce visible-light photocatalysis on the surfaces of wide band-gap semiconductors, such as TiO_2_ [32]. Chlorella is rich in β-carotene and chlorophyll. When these dyes are adsorbed on TiO_2_ through the weak Van der Waals interaction, TiO_2_ becomes sensitized which raises its photocatalytic activity. Before the hydrothermal process, two important factors are found to influence the photocatalytic performance. Firstly, TiO_2_ only exhibits the amorphous phase, which leads the lower photocatalytic activities. Secondly, most photosynthesis pigments (PBPs) cannot be released from the Chlorella cells to combine with TiO_2_, due to the limitation of the cell walls. The schematic diagram of visible-light activation of TiO_2_ by dye sensitization is shown in Figure 9. Under the visible-light illumination, the adsorbed dye molecules on the surface are excited and electrons are injected into the CB of TiO_2_ [32]. The electrons injected by the dye molecules are then quickly transferred to the surface of TiO_2_. The electrons can reduce the oxygen absorbed on the catalyst to superoxide radical O^2−^, which is the reactive species responsible for the degradation of RhB [33,34].

## 4. Conclusions

A hydrothermal method was used to immobilize TiO_2_ nanoparticles on *Chlorella pyrenoidosa* cells. Compared with the pure TiO_2_, the obtained chlorella/TiO_2_ composite has larger average sizes and higher absorbance in the visible-light region. The chlorella/TiO_2_ composite exhibits enhanced photocatalytic activity towards the degradation of RhB dye under visible-light irradiation. The likely reaction mechanism is linked to the highly efficient charge separation of electron/hole pairs due to the sensitization of anatase TiO_2_ with the photosynthesis pigments of chlorella cells.

## Supplementary Materials

The following are available online at www.mdpi.com/1996-1944/10/5/541/s1. Figure S1: Optical images of the chlorella cells before and after hydrothermal treatment, Figure S2: Particle size distribution of the chlorella/TiO_2_ composite and pure TiO_2_, Figure S3: Ti 2p XPS spectra of the two analyzed samples, Table S1: The C 1s and O 1s contents in the samples, extracted from the XPS data, Figure S4: XRD patterns of the chlorella/TiO_2_ composite and pure TiO_2_ before and after the hydrothermal treatment, Figure S5: Photocatalytic degradation of RhB solution with the samples without the hydrothermal treatment under visible-light illumination.
