# Supplementary material for: Immobilization of TiO2 Nanoparticles on Chlorella pyrenoidosa Cells for Enhanced Visible-Light-Driven Photocatalysis
Source: Materials (Basel). 2017 May 17;10(5):541. doi: 10.3390/ma10050541 (PMC5459020; doi:10.3390/ma10050541)
Supplement: Supplementary file 1 [file materials-10-00541-s001.pdf]

Immobilization of TiO_2 Nanoparticles on *Chlorella pyrenoidosa* Cells for Enhanced Visible-Light-Driven Photocatalysis

Aijun Cai [1,2], Aiying Guo [1] and Zichuan Ma [2,*]

[1] College of Life Science and Technology, Hebei Normal University of Science & Technology, Qinhuangdao 066600, PR China; 2755@hevttc.edu.cn (A.C.); 2695@hevttc.edu.cn (A.G.)

[2] College of Chemistry and Material Sciences, Hebei Normal University, Shijiazhuang 050016, PR China

* Correspondence: mazc@hebtu.edu.cn; Tel.:+86-311-80787402

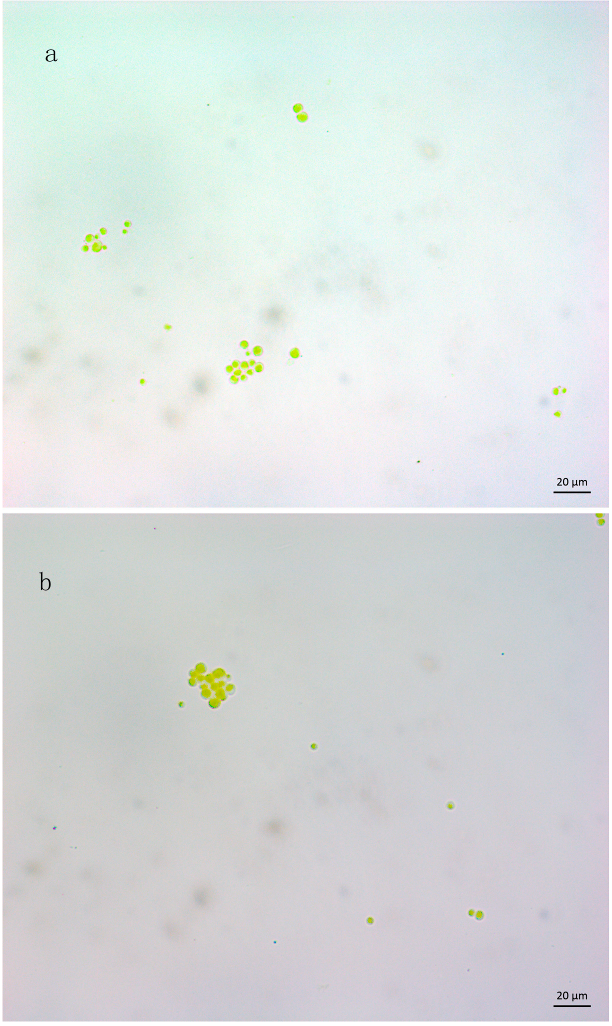

Figure S1. Optical images of chlorella cells (**a**) before and (**b**) after the hydrothermal treatment.

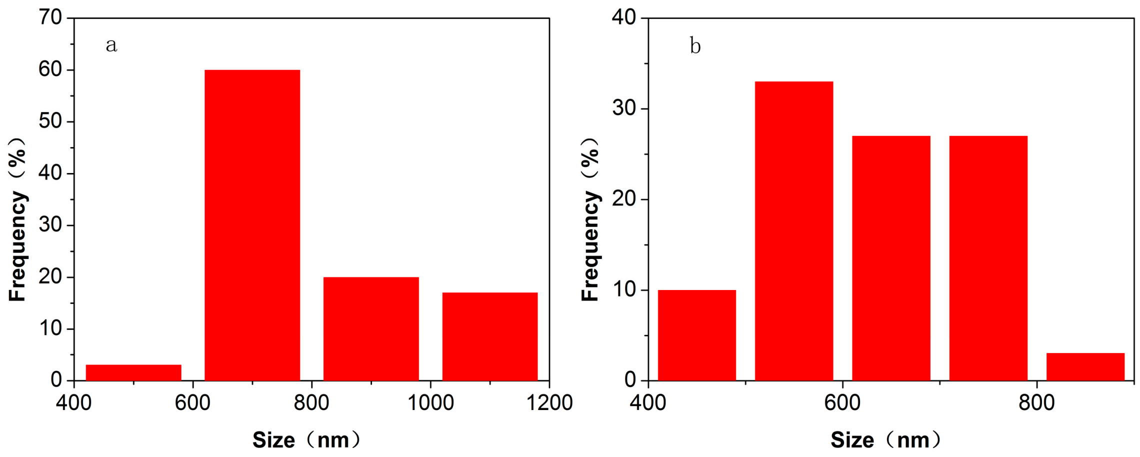

Figure S2. Particle size distribution of the chlorella/TiO_2 composite (**a**) and pure TiO_2 (**b**).

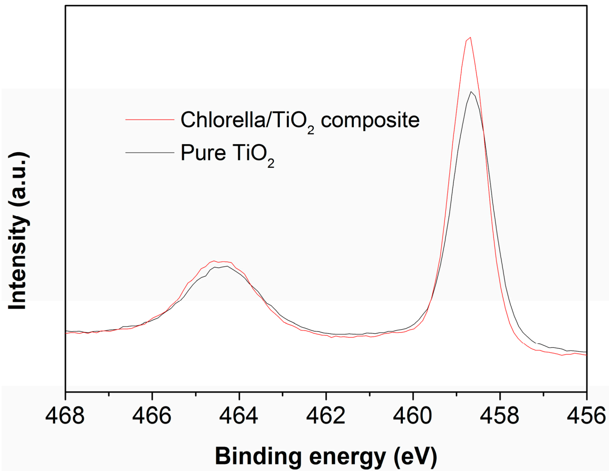

Figure S3. Ti 2p XPS spectra of the two analyzed samples.

Table S1. The C 1s and O 1s contents in the samples, extracted from the XPS data.

Samples	Elements	PPAt.%
Pure TiO_2	C1s	39.77
Chlorella/TiO_2 composite	C1s	46.86

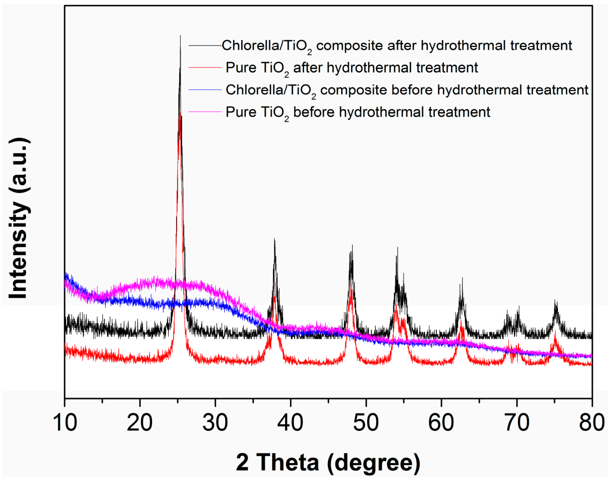

Figure S4. XRD patterns of the chlorella/TiO_2 composite and pure TiO_2 before and after the hydrothermal treatment.

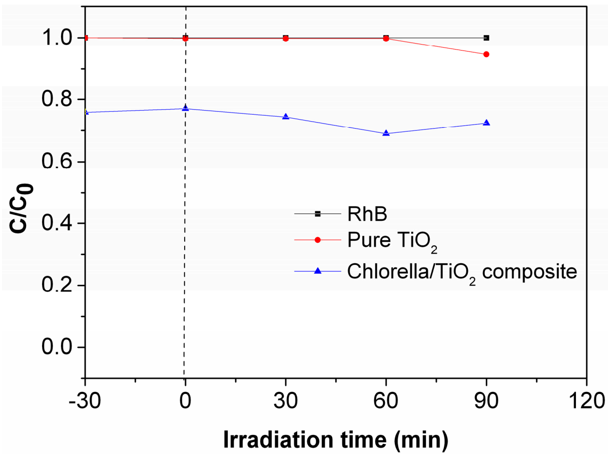

Figure S5. Photocatalytic degradation of RhB solution with the samples without the hydrothermal treatment under visible-light illumination.

© 2017 by the authors. Submitted for possible open access publication under the terms and conditions of the Creative Commons Attribution (CC-BY) license (http://creativecommons.org/licenses/by/4.0/).
